# Feature and motion-based gaze cuing is linked with reduced social competence

**DOI:** 10.1038/srep44221

**Published:** 2017-03-10

**Authors:** Dana A. Hayward, Jelena Ristic

**Affiliations:** 1 Department of Psychology, Concordia University, Montréal, QC, Canada; 2 Department of Psychology, McGill University, Montréal, QC, Canada.

## Abstract

Gaze following is a fundamental ability that plays an important role in human social function. However, the link between these two processes remains elusive. On the one hand, typically developing persons show robust gaze following in laboratory cuing tasks. On the other hand, investigations with individuals with autism suggest that reduced social competence in this population may partly reflect an atypical access to social information through attending to perceptual changes that normally accompany gaze shifts, like luminance or motion transients. Here we investigated if gaze cuing in typically developing individuals was modulated by similar task-irrelevant perceptual changes. In Experiment 1, a social gaze cue was presented with or without a luminance change. In Experiment 2, a social gaze cue was presented together with a motion cue. Both experiments indicated reduced magnitudes of gaze cuing in persons with low social competence on trials containing an irrelevant perceptual change. This suggests that similarly to individuals with autism, typically developing persons with low social competence also utilize idiosyncratic perceptual changes in the environment to access social content, revealing strong links between basic gaze following abilities and a range of social competence within typical individuals.

Spontaneous gaze following is a fundamental socio-cognitive process that facilitates social communication and furnishes typical social competence (i.e., the effectiveness of forming and maintaining social relationships^1^). Often, gaze following is studied by quantifying naturalistic interactions^2^,^3^,^4^; however during recent decades it has also been operationalized in laboratory procedures like the cuing paradigm, which facilitate experimental control and enable a precise quantification of gaze following behavior^5^,^6^ and its underlying mechanisms^7^,^8^.

While the application of controlled experimental procedures like the cuing task has generated a wealth of knowledge about how humans perceive and follow gaze cues^9^,^10^,^11^,^12^, the question of how gaze cuing relates to real world social behavior has not yet received much research consideration. This is because most investigations conducted to date^6^,^9^,^13^,^14^ have examined gaze cuing in typically^9^ and atypically developing participants (i.e., individuals with high functioning autism^15^,^16^) as a group regardless of the participants’ individual level of social function.

The main goal of the present study was to examine how gaze cuing varied as a function of social competence within the typical population. More specifically, we were interested in assessing whether level of social competence was associated with how participants perceived and attended to social cues. To do so we manipulated both the gaze cues and task-irrelevant perceptual changes and assessed how the resultant gaze cuing magnitude varied as a function of a broad measure of social competence in which we included stable indices of gender^17^ and number of autistic-like traits^18^, as well as more dynamic indices reflecting the extent of participants’ real^19^ and perceived social networks^20^. The data from our two experiments indicated that individuals low in social competence attended to social cues differently than individuals high in social competence. Specifically, the presence of task-irrelevant perceptual changes within the cuing task resulted in reduced magnitudes of gaze cuing in individuals low in social competence, a finding that resembles the data reported with persons with autism^21^,^22^.

## Gaze Cuing at the Group Level

Past investigations that have examined gaze cuing in healthy participants reliably show that humans shift their attention in response to gaze direction spontaneously and automatically. This finding is frequently revealed using a modified version of the Posner cuing task^23^ in which pupils, acting as an attentional cue, appear within a face and participants respond to target events that appear either at the gazed-at (i.e., valid) or not gazed-at (i.e., invalid) locations. Typically, better performance (e.g., target detection, localization, or discrimination) for valid relative to invalid targets is found (see ref. 9 for a review) even when gaze direction does not provide any pertinent information about the target’s location^5^,^24^.

This so-called ‘gaze cuing’ effect is thought to reflect the underlying workings of the human gaze following mechanism by enabling quick and direct access to social information in the environment. As such, this basic behavior is often interpreted theoretically as being a precursor for the development of more complex social abilities like social communication^25^, theory of mind^26^, and relationship formation^27^. Consistent with this idea, gaze cuing is observed early in development^6^,^28^,^29^,^30^, depends on right-lateralized brain structures specialized for eye direction computation^31^,^32^, and generally shows resilience to contextual factors like the complexity of the face stimulus (e.g., photographs of real vs. schematic faces^24^,^33^,^34^).

## Individual Differences in Gaze Cuing

Although the literature firmly indicates the presence of a reliable overall, group-level gaze cuing effect within the typical population^24^,^35^,^36^,^37^,^38^, the few studies that have examined individual variability in this effect have shown that the magnitude of gaze cuing (i.e., the performance difference between valid and invalid trials), which reflects the relative strength of the social gaze cue to engage one’s attention, varies with participants’ gender^17^,^39^,^40^ and their number of autistic-like traits^17^, AQ^18^,^41^,^42^,^43^. Bayliss and colleagues^17^ were among the first to report that females and individuals with fewer autistic-like traits showed larger gaze cuing, with no similar individual variability found in nonsocial cuing effects (i.e., when peripheral onsets served as attentional cues).

## Gaze Cuing in Autism

These data with normative participants predict that gaze cuing should be atypical in individuals with autism who exhibit dysfunctions in everyday social function^44^. Surprisingly, however, most investigations conducted to date have failed to support this notion, reporting equivalent gaze cuing between individuals with autism and typically developing peers^16^,^28^,^45^,^46^,^47^,^48^,^49^,^50^,^51^,^52^. For example, Swettenham *et al*.^48^ found no differences in gaze cuing between individuals with autism and matched controls in an experiment that utilized a gaze cue in which the pupils shifted from looking straight ahead to a left or right deviated position (see also ref. 45). No differences between clinical and control groups were also reported by Senju *et al*.^16^, who measured gaze cuing in response to photographs of faces which at first displayed closed eyes followed by averted eyes before being abruptly extinguished altogether at the time of target presentation.

Ristic *et al*.^15^ proposed that this oft-reported equivalence in gaze cuing between typical participants and those with autism reflects the difference by which individuals with autism and typically developing persons access the social information conveyed by a gaze cue (e.g., intentions or desires of others^53^). According to their ‘feature correspondence hypothesis’, individuals with autism utilize perceptual feature changes (e.g., motion or visual transients^23^,^54^,^55^,^56^,^57^) that often accompany gaze shifts to align their attention with the direction indicated by a gaze cue. While this results in experimental effects that mirror typical gaze cuing, orienting of attention is elicited by perceptual rather than social information, and leads to compromised or reduced access to the social content.

At least two lines of evidence support this idea. First, in the majority of gaze cuing studies which have found equivalent effects between clinical and typical populations, a gaze cue was presented in conjunction with a simple visual change, like an abrupt visual transient or pupil motion^16^,^28^,^45^,^46^,^47^,^48^,^49^,^50^. It is well known that such perceptual changes represent powerful attentional cues^23^,^54^,^55^,^56^,^57^, and that persons with autism orient their attention to those stimuli normatively if not preferentially^58^,^59^. Second, there is a growing emphasis on understanding the role of atypical perceptual processing abilities in the manifestation of social deficits observed in the autism spectrum. Relative to typically developing peers, individuals with autism have an increased number of connections within the primary visual cortical areas^60^,^61^,^62^ and exhibit reduced connectivity between the primary visual area and higher level extrastriate visual cortices^63^ as well as frontal regions^64^. Recent prospective longitudinal studies suggest that such architectural atypicalities manifest early in behavior, and are linked with severity of the emerging ASD phenotype at later ages^64^. As such, these underlying perceptual preferences may afford participants with autism particular sensitivity to simple perceptual changes over social information.

In contrast, typically developing persons are hypothesized to prioritize social over perceptual information^53^,^65^,^66^. Ristic *et al*.^15^ named this process the ‘social reading hypothesis’, arguing that in typical development the social content of gaze is immediately and directly accessed irrespective of any additional coincidental perceptual information.

Given these differences in processing, persons with atypical development in the social domain may bootstrap the social content from simple visual changes that often accompany changes in social cues (e.g., see refs 67, 68, 69 for similar findings in early typical development) while those developing typically access the cues’ social content directly. In turn, these perceptual preferences may relate not only to disorders in social function, like autism, but also to the level of social competence within the typical population e.g. refs 70 and 71.

## Experiment 1

Following this, we reasoned that gaze cuing in typically developing participants low in social competence might be affected by concurrent low-level perceptual changes in the environment. To dissociate the feature correspondence and the social reading hypotheses, in Experiment 1 we manipulated luminance transients independently and orthogonally from social gaze information. This allowed us to assess any influence of a perceptual change on the gaze cuing effect, while at the same time providing a clear measure of gaze cuing devoid of low-level confounding artifacts. We predicted that individuals low in social competence would show decreased magnitudes of gaze cuing when a perceptual change was present in the task relative to when it was absent. In contrast, gaze cuing in individuals high in social competence should be resilient to such perceptual fluctuations.

To get a broad assessment of individual differences in social competence, we analyzed variations in gaze cuing performance not only as a function of participant’s gender and their Autism Spectrum Quotient scores (AQ^18^), but also as a function of their dynamic real world social behaviors as reflected by estimates of actual social network (Social Network Questionnaire (SNQ)^19^) and perceived social network sizes (number of facebook (FB) friends from the Social Network Size Questionnaire (SNSQ)^20^). We included the last two measures for two reasons. One, past research has shown that individuals reporting greater number of social conversations and/or number of Facebook friends also showed increased grey matter volume and/or density in various brain areas associated with socio-cognitive processes, such as the superior temporal sulcus (STS)^19^,^20^. Two, individuals reporting larger numbers of Facebook friends have also been found to exhibit increased psychological well-being^72^ as well as stronger perceptions of social support, reduced stress, and fewer instances of physical illness^73^. Including all of these measures provided us with indices of both stable and dynamic individual social characteristics and facilitated cross-level comparisons between gaze cuing and a broadly defined construct of social competence.

## Methods

### Participants

Sixty-four undergraduate students (32 males; mean age = 20.4, SD = 2.7) were recruited via volunteer participant pool, and were compensated with course credits. All experimental procedures were approved by the McGill University Research Ethics Board and adhere to the principles of the Helsinki Declaration. Written informed consent was obtained from all participants.

### Apparatus and Stimuli

**Figure 1:**
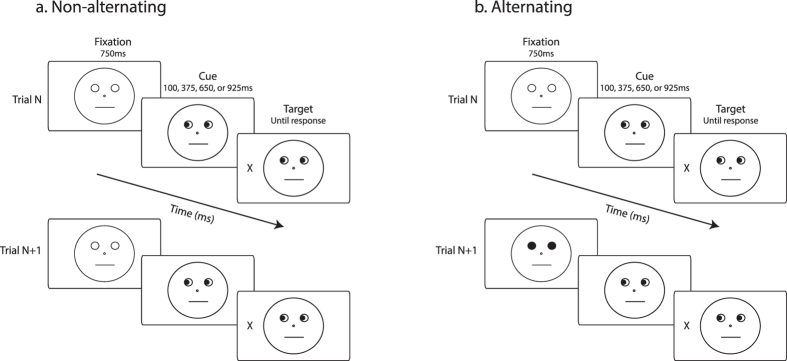
Experiment 1 Stimulus Presentation Sequence. Each trial started with a fixation screen, which depicted either a blank face with white eye outlines (Fig. 1a) or a blank face with black filled-in eye outlines (Fig. 1b, Trial N + 1). After 750 ms, black pupils looking either to the left or right appeared for 100, 375, 650 or 925ms before the response target was presented on either the left or right side. Participants were asked to detect the target’s onset as quickly as possible by pressing the spacebar. Figure 1a illustrates a *non-alternating* trial (i.e., Trial N + 1), in which the same fixation display type was presented on two consecutive trials. Figure 1b illustrates an *alternating* trial (i.e., Trial N + 1), in which the fixation display type changed between two consecutive trials. Stimuli are not drawn to scale.

The stimuli and example trial sequences are illustrated in Fig. 1. As in past studies^24^, we elicited gaze cuing using a schematic face. The face was comprised of a circle outline (measuring 9.4° of visual angle), pupils (0.7°), depicted by black filled-in circles shown within eye outlines (1.1°), mouth (3°), depicted by a horizontal line, and a nose (0.3°), depicted by a small center circle. The response target was a capital letter ‘X’ (1°), presented 7° away from central fixation along the horizontal meridian.

### Design

A within-subjects design with fixation type, gaze cue validity, and cue-target time was used. All variables were presented equally often in a random intermixed fashion.

The perceptual change was manipulated by alternating the fixation display randomly between an image depicting a face with white filled-in eye outlines and an image depicting a face with black filled-in eye outlines. *Non-alternating* trials (Fig. 1a) contained no perceptual change from one trial to the next. That is, two consecutive trials showed the same fixation display (either white filled-in eye outlines or black filled-in eye outlines). In contrast, *alternating* trials (Fig. 1b) contained a perceptual change from one trial to the next. That is, two consecutive trials showed different fixation displays (e.g., a trial with the fixation display showing black filled-in eye outlines preceded by a trial showing the fixation display with white filled-in eye outlines, or vice versa). This perceptual change occurred in approximately one half of trials (alternating trials = 50.2%) and both fixation display types were equally likely to precede a valid or an invalid trial.

Gaze cuing was always elicited using black filled-in pupils. On any given trial, the gaze cue could either be valid (i.e., pupils gazing at the target) or invalid (i.e., pupils gazing away from the target) with respect to the target location. The gaze cue did not provide any reliable spatial information about the location of the target (i.e., p = 0.5) and indicated a left or right target location equally often. The target was equally likely to occur at a left or right location.

Cue-target time, or the time between the presentation of the gaze cue and the presentation of the target was manipulated to understand the temporal profile of gaze cuing and varied randomly between 100, 375, 650, and 925 ms.

Thus, rather than presenting the perceptual change in conjunction with the gaze cue, we manipulated the perceptual change orthogonally and randomly between trials while keeping the gaze information constant within trials. This enabled us to examine the effects of a changing perceptual environment on the concomitant gaze cuing effects.

### Social Competence Measures

Participants’ scores on the three questionnaires were used to assess social competence. The AQ^18^ reflects the number of autistic-like traits in the typical population, with higher scores denoting the presence of more autistic-like traits. We also evaluated the extent to which participants interacted with others using two measures of social network, the SNQ and FB. The SNQ^19^ yields a measure of one’s social network size by quantifying the number of individuals a participant has had personal contact or communication with that was primarily social in nature within the last month. Perceived network size was assessed by asking participants to report their number of friends on Facebook (FB^20^). Thus, while the AQ indicated more stable social traits, the SNQ and FB returned more dynamic measures of social functioning in day-to-day life. Questionnaires were administered in a randomized order after the experimental procedure was completed.

### Procedure

As illustrated in Fig. 1, all trials began with the presentation of a fixation display showing a face outline with either white or black filled-in eye outlines for 750 ms. Next the gaze cue, indicating either a left or right direction was revealed. After a variable time interval, the target demanding a detection response appeared on the left or right of fixation. The gaze cue and the target remained on the screen until a response was made or 2000 ms had elapsed. Approximately 6% of trials contained no target. Response Time (RT) was measured from target onset.

Participants viewed the task on a 16-in CRT monitor from an approximate distance of 57 cm. Before the task, the types of stimuli, the task sequence, and the task were explained to the participants. They were told that the direction of gaze did not predict the location of the target. They were instructed to press the spacebar as quickly and as accurately as possible in response to the target, and to refrain from responding on no-target trials. A total of 612 trials, divided equally across three testing blocks were run. Five practice trials were run at the start. All participants were naïve to the purpose of the study and were fully debriefed after the session.

## Results

RTs were examined for anticipations (i.e., RTs < 100 ms), timeouts (i.e., RTs > 1000 ms), and false alarms (i.e., responding on no-target trials). All types of errors were infrequent, with anticipations and timeouts accounting for 2.7% and false alarms for 1.3% of data. Errors were removed from all analyses.

To ensure that the type of fixation display (black vs. white filled-in eye outlines) did not influence overall RTs, we ran an omnibus repeated measures ANOVA with fixation type, cue validity (valid vs. invalid), and cue-target interval (100, 375, 650, and 925 ms). The analysis returned a reliable overall gaze cuing effect [F(1,63) = 24.4, p < 0.001], which was not modulated by fixation type, i.e., there were no main effects (F < 1) or interactions involving fixation type [fixation type x cue validity, F < 1; fixation type x cue validity x cue-target interval, F < 1.5, p > 0.2; all other Fs < 1.5, all ps > 0.3].

We performed the following analyses to evaluate our hypotheses. First, we inspected gaze cuing at the group level as a function of non-alternating and alternating trials. Then, we examined if gaze cuing during non-alternating and alternating trials varied as a function of gender and average social competence. Finally, using regression analyses we investigated if the individual participants’ magnitudes of gaze cuing for non-alternating and alternating trials varied as a function of gender and individual scores on the AQ, SNQ, and FB measures.

### Group-Level Analyses

**Figure 2:**
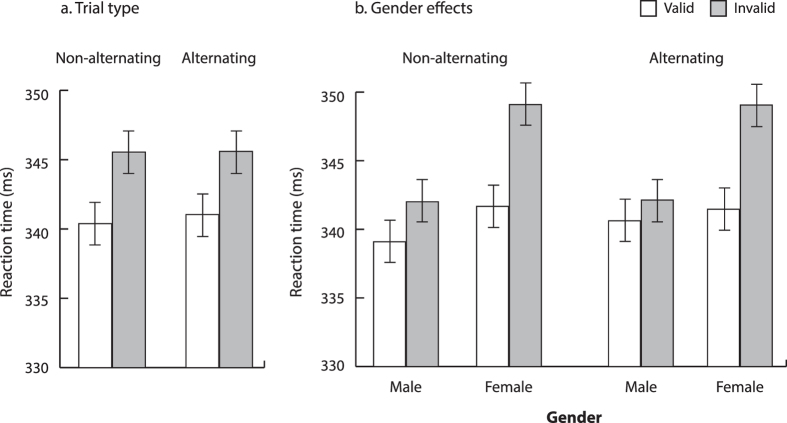
Experiment 1 Group-based RT Results. Figure 2a shows mean interparticipant correct RTs as a function of trial type (i.e., non-alternating vs. alternating) and gaze cue validity. Figure 2b shows mean interparticipant correct RTs as a function of trial type, gender, and gaze cue validity. Error bars depict standard error of the difference between the means.

To analyze if gaze cuing varied as a function of perceptual change during non-alternating and alternating trials, we computed interparticipant mean correct RTs as a function of the previous trial’s fixation display type. These means were subjected to a repeated measures ANOVA with trial type (non-alternating vs. alternating), cue validity (valid vs. invalid), and cue-target interval (100, 375, 650, and 925 ms). Replicating past research^9^, our results shown in Fig. 2a indicated reliable overall gaze cuing [cue validity; F(1,63) = 24.3, p < 0.001] with faster responses for valid relative to invalid targets. Participants’ responses became faster as the cue-target interval increased [F(3,189) = 211.1, p < 0.001], reflecting the typical foreperiod effect^74^. No interactions were significant (all Fs < 1.2, all ps > 0.3).

Furthermore, and replicating Bayliss *et al*.^17^, reduced gaze cuing was found in male relative to female participants, as shown in Fig. 2b. This observation was confirmed by including gender as a between-subjects variable in the preceding ANOVA, which returned a reliable gender x cue validity interaction [F(1,62) = 8.0, p < 0.01] and no additional effects or interactions involving gender (all Fs < 2.5, ps > 0.05). Separate ANOVAs conducted for each gender group supported this conclusion, returning a statistically significant gaze cuing effect for females [F(1,31) = 28.4, p < 0.001] and a marginal gaze cuing effect for males [F(1,31) = 3.2, p = 0.08].

Thus, when we examined the data using conventional group-based analyses, we replicated a large body of evidence showing group-level gaze cuing^5^,^9^,^24^,^34^,^36^ which overall did not vary with perceptual changes in the display but indicated reduced gaze cuing effects in male participants.

### Average Social Competence

Next, we examined the link between gaze cuing and social competence. Data were inspected for outliers prior to all correlation and regression analyses. Outliers were defined as any score exceeding three standard deviations from the group mean. This resulted in the exclusion of one data point.

The Pearson correlation analyses indicated that the scores on the three scales related predictably, as shown in Fig. 7, which illustrates questionnaire correlations for both Experiment 1 and Experiment 2. Specifically, for Experiment 1, participants’ scores on the AQ were related negatively to their scores on both the SNQ and FB (r = −0.433, p < 0.001; r = −0.347, p < 0.01), with an increased number of autistic-like traits associated with a smaller social network. Scores on the two measures of social network also related predictably, with an increase in real social network size (SNQ) accompanied by an increase in perceived social network size (FB, r = 0.273, p < 0.05).

To remind, we hypothesized that individuals low in social competence would show decreased gaze cuing magnitudes when a perceptual change was present (i.e., during alternating trials) relative to when it was absent (i.e., during non-alternating trials). To test this, we analyzed if the gaze cuing effects for non-alternating and alternating trials varied as a function of participants’ average level of social competence, as determined by a median split on a composite social competence score derived from all questionnaires. The composite score was calculated using a procedure reported in Dodd, Hibbing, and Smith^75^. This technique permits a composite analysis of multiple questionnaire data using a within-questionnaire quartile score breakdown. It yields an aggregated individual score in which each questionnaire contributes equally to the composite measure. To compute the composite score, first, individual scores on each questionnaire (i.e., AQ, SNQ, and FB) were assigned a quartile rank within that questionnaire. The quartile rank ranged from 1 (here indexing low social competence) to 4 (indexing high social competence). AQ was reverse-scored, given that high scores on this questionnaire yield a high number of autistic-like traits, i.e., low social competence. Table 1 summarizes AQ, SNQ, and FB scores as a function of quartile score ranks. Next, the quartile ranks for each questionnaire were summed for each participant. This yielded a composite social competence score for each participant. In our sample, the composite scores ranged from 3 to 12, with low numbers denoting low social competence and high numbers denoting high social competence. Participants with composite social competence scores falling between 3 and 7 were classified as the low social competence group (N = 32; 19 males). Participants with composite social competence scores falling between 8 and 12 were classified as the high social competence group (N = 32; 13 males).

**Figure 3:**
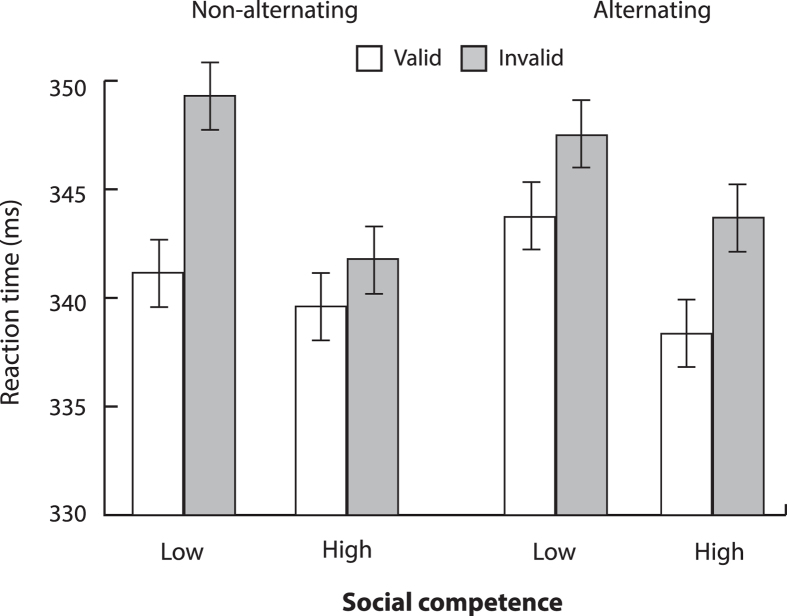
Experiment 1 RT Data as a Function of Average Social Competence. Figure 3 shows mean interparticipant correct RTs as a function of average social competence, trial type (i.e., non-alternating vs. alternating), and gaze cue validity. Error bars depict standard error of the difference between the means.

To analyze if gaze cuing for non-alternating and alternating trials varied as a function of average level of social competence, we performed a mixed effects ANOVA with social competence group (low vs. high) included as a between-subjects factor, and trial type (non-alternating vs. alternating), cue validity, and cue-target interval included as within-subjects factors. The data once again revealed main effects of gaze cuing [cue validity; F(1,62) = 24.4, p < 0.001] and cue-target interval [F(3,186) = 209.7, p < 0.001]. Critically however, gaze cuing interacted with group and trial type [group x trial type x cue validity; F(1,62) = 7.4, p < 0.01] suggesting that gaze cuing magnitude during alternating trials was reduced in individuals low in social competence, as illustrated in Fig. 3. This stood in contrast to individuals high in social competence, who showed steady gaze cuing effects for both non-alternating and alternating trials. No other effects or interactions reached significance (all Fs < 1.5, ps > 0.2).

One might wonder if the interaction between social competence, trial type, and cue validity was driven by a reduction in gaze cuing from non-alternating to alternating trials for individuals low in social competence or by an increase in gaze cuing from non-alternating to alternating trials for individuals high in social competence. To examine the directionality of this three-way interaction, we analyzed the data from the two groups separately using repeated measures ANOVAs, each including trial type, cue validity, and cue-target interval as factors. The results supported the first alternative. Both analyses returned significant main effects of cue validity [high social competence group, F(1,31) = 6.5, p < 0.05; low social competence group, F(1,31) = 20.9, p < 0.001], indicating present gaze cuing effects in each group. However, a sole interaction between previous trial type and cue validity [F(1,21) = 6.7, p < 0.05] emerged in the low social competence group only, indicating a reduction in the gaze cuing magnitude for alternating trials in individuals low in social competence (8.1 ms vs. 3.8 ms; all other Fs < 1, all ps > 0.3). A follow-up paired t-test, comparing magnitudes of gaze cuing (i.e., average invalid RT – average valid RT) between the non-alternating and alternating trials confirmed decreased gaze cuing for the alternating trials (t(31) = 2.6, p < 0.05). No interactions were reliable in the high social competence group (all Fs < 1.6, all ps > 0.19).

### Individual Differences in Social Competence

These group results suggest that participants’ questionnaire scores along with their gender should account for a reliable amount of variability in the magnitudes of the gaze cuing effect during non-alternating and alternating trials. The data from two multiple regression analyses confirmed this hypothesis.

**Figure 4:**
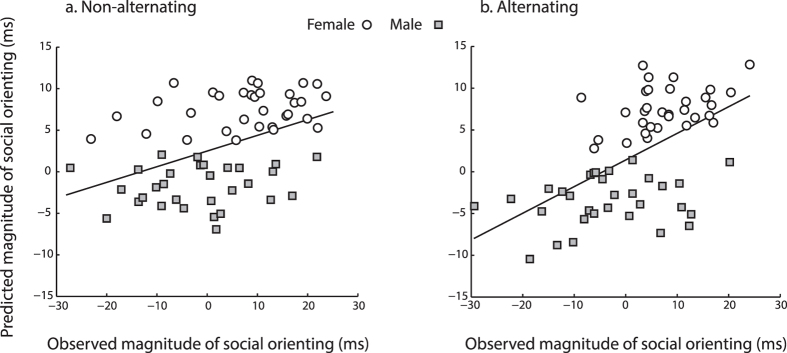
Experiment 1 Regression Results. Figure 4 shows the scatterplots displaying the relationship between individual participants’ observed (x-axis) and predicted (y-axis) magnitudes of gaze cuing, for the non-alternating (**a**) and alternating (**b**) trials. Participants’ gender is marked by circle (Female) and square (Male) data points.

Gaze cuing effect magnitude was calculated by subtracting each participant’s overall mean RT on valid trials from their mean RT on invalid trials separately for non-alternating and alternating trials. Then, participant’s gender and their raw AQ, SNQ, and FB scores were entered as predictor variables of the magnitude of the gaze cuing effect for non-alternating and alternating trials separately. Figure 4 shows the two regression models, plotting the relationship between the participants’ observed magnitudes of gaze cuing (x-axis) and their predicted magnitudes of gaze cuing (y-axis) for non-alternating (4a) and alternating (4b) trials. Both models were statistically reliable, however the model for the alternating trials had a better fit overall, in that about 30% of the variability in the magnitudes of gaze cuing was accounted for by the predictor variables [Alternating: R^2^ = 0.28, F(4, 58) = 7.0, p < 0.0001; Non-alternating: R^2^ = 0.12, F(4, 58) = 3.2, p < 0.05]. Specifically, the model for alternating trials indicated that participants’ gender and their AQ were significant predictors [gender: β = −0.488, t = −4.2, p < 0.001; AQ: β = −0.300, t = −2.4, p < 0.05], while the semipartial correlation coefficients further indicated gender as a stronger predictor than AQ, accounting for 20.3% (vs. 6.6% for AQ) of the variance. The regression model for non-alternating trials indicated only gender as a reliable predictor of the gaze cuing magnitude [β = −0.398, t = −3.1, p < 0.01].

## Discussion

In Experiment 1 we examined if gaze cuing varied as a function of social competence and task-irrelevant luminance changes. Our results indicated overall group-level gaze cuing effects, replicating a large body of existing research^5^,^9^,^24^,^34^,^36^. However, when we examined the data as a function of participants’ social competence by using both a median-split composite score and individual participants’ scores, we found that participants who scored low on our broad measure of social competence displayed reduced gaze cuing effects when the task contained a luminance change. This stands in contrast to participants who scored high on social competence measures, whose gaze cuing effects were unaffected by such perceptual fluctuations. These data support the idea that individuals with low social competence may utilize coinciding low-level task-irrelevant visual transients in the environment to access social content from social cues. In Experiment 2, we assessed whether a similar result is obtained when instead of a luminance change the gaze cue is presented alongside an apparent motion cue.

## Experiment 2

Stimulus motion remains one of the most powerful attentional cues^55^,^76^,^77^,^78^. As such, it is thought to play an important role in the development of social orienting. Typically developing infants first start to orient their attention in response to the low level perceptual changes that accompany gaze shifts (luminance^69^, motion^67^) before starting to access social information conveyed by the gaze cue implicitly at about 4 months of age and explicitly at about 3 years of age^68^,^69^. While many studies that have examined gaze cuing have presented gaze cues in conjunction with pupil motion, to the best of our knowledge, only one study conducted so far has assessed the contributing effect of such motion cues on gaze cuing in adults^17^. This investigation did not find any modulating role of motion on the gaze cuing effect.

**Figure 5:**
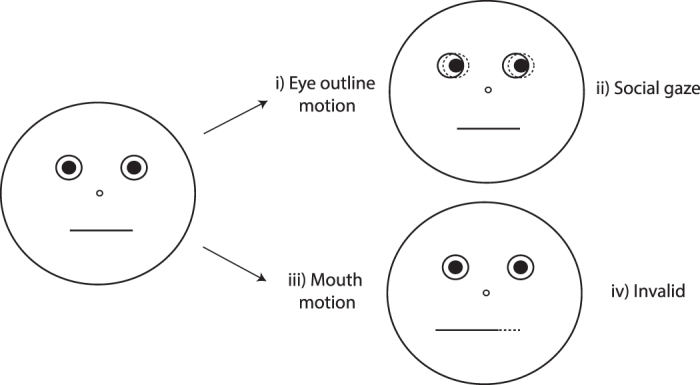
Experiment 2 Trial Types. Figure 5 illustrates the four trial types resulting from the displacement of either the eye outlines or the mouth line: (i) *Eye outline motion*, (ii) *Social gaze*, (iii) *Mouth motion*, and (iv) *Invalid.* Stimuli are not drawn to scale.

In Experiment 2, we presented participants with a gaze cue and an opposing motion cue. This was accomplished in two ways. First, and as shown in Fig. 5, on one half of trials, we displaced the eye outlines of a straight gaze cue to the left or right, creating an apparent motion transient towards either a left or right peripheral location. Because the pupils remained centered, displacing the eye outlines resulted in the perception of deviated gaze in the opposite direction, creating opposing eye outline motion and social gaze cues. Second, on the other half the trials, we shifted the mouth line to either the left or right, again creating an apparent motion transient towards either a left or right peripheral location. Here, only the mouth line was displaced, allowing for the examination of motion cuing effects alone. Like Experiment 1, this design dissociates the feature correspondence and the social reading hypotheses by manipulating the motion cue orthogonally and independently from social gaze direction.

## Methods

### Participants

Sixty-four new naïve participants (32 males; mean age = 22.6, SD = 3.8) were recruited using the same means as in Experiment 1. All experimental procedures were approved by the McGill University Research Ethics Board and adhere to the principles of the Helsinki Declaration. Written informed consent was obtained from all participants.

### Apparatus, Stimuli, Design and Procedure

Experiment 2 utilized the same apparatus, stimuli, and the general stimulus presentation sequence as Experiment 1. A within-subjects design, with trial type (i.e., Eye outline motion, Social gaze, Mouth motion, or Invalid) and cue-target time (100, 375, 650 or 925 ms) was used.

Figure 5 illustrates the Experiment 2 cuing conditions. After the presentation of a fixation screen showing straight gaze, either the two eye outlines or the mouth line were displaced laterally by 2.1° relative to centre. This amount of physical displacement ensured that the end of the displaced mouth line aligned with the outer edge of the displaced eye outline. After 100, 375, 650 or 925 ms, the target demanding a detection response appeared on either the left or right side of the face.

There were four trial types. *Eye outline motion* trials were those in which the target appeared at the location cued by the eye outline motion; *Social gaze* trials were those in which the target appeared at the location cued by gaze; *Mouth motion* trials were those in which the target appeared at the location cued by mouth motion; *Invalid* trials were those in which the target appeared at the location cued by none of the other three cues. Each combination of cue type (eye outline; mouth), cue displacement (left; right), target position (left; right), and cue-target time was presented equally often in a pseudorandom order. Thus, as in Experiment 1, the cues were fully uninformative about the location of the target.

Likewise, participants were instructed about the stimuli, task sequence, and the task. They were told the cues were fully nonpredictive about the target location, asked to maintain central fixation, and to respond as fast and as accurately as possible by pressing the spacebar. Each participant completed a total of 612 trials divided evenly across three testing blocks. Approximately 6% of trials contained no target. Five practice trials were run at the start. Each participant completed the AQ, SNQ, and FB questionnaires after the experimental task in random order. Participants were fully debriefed afterwards.

## Results

Response errors (i.e., response anticipations, time outs, and false alarms) were rare and removed from all analyses. Anticipations and timeouts accounted for 0.9% and false alarms for 1.6% of the data.

Once again, three types of analyses were conducted. First, to examine the effects of motion on gaze cuing, we conducted ANOVAs for trials on which the gaze cue competed with the motion cue (Social Gaze vs. Eye Outline Motion) and separately for trials on which the motion cue occurred by itself (Mouth Motion vs. Invalid), overall and as a function of gender. Then, we analyzed if those effects varied with participants’ average and individual level of social competence.

### Group-level Analyses

**Figure 6:**
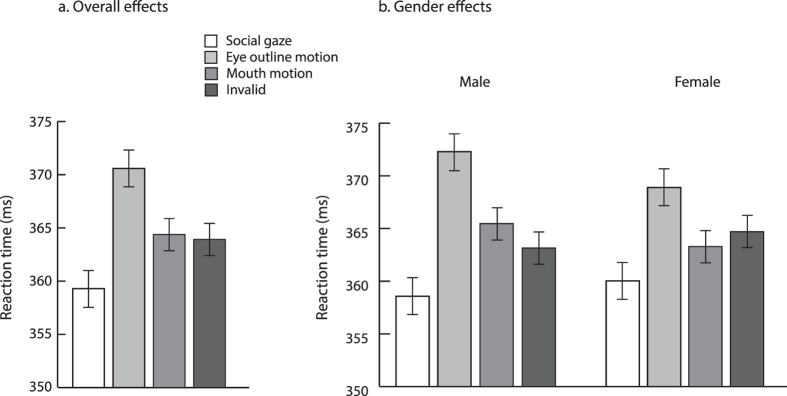
Experiment 2 Group-based RT Results. Figure 6a shows mean interparticipant correct RTs as a function of trial type (i.e., Social eyes, Eye outline motion, Mouth motion, Invalid). Figure 6b shows mean interparticipant correct RTs as a function of trial type and participants’ gender. Error bars depict standard error of the difference between the means.

**Figure 7 f7:**
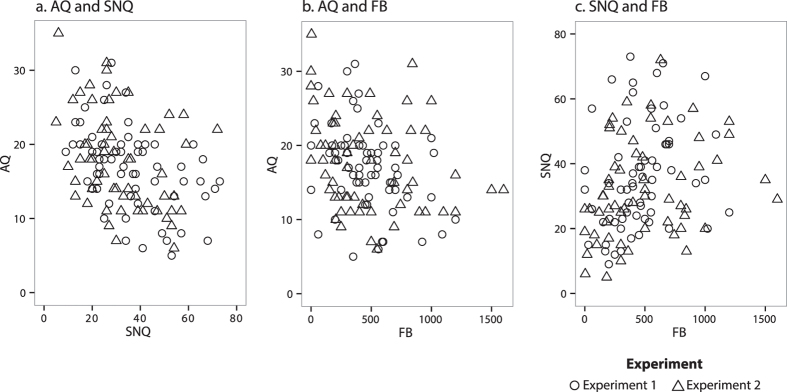
Relationship Between Questionnaire Measures. Figure 7 shows the scatterplots indicating the relationship between participants’ scores on the AQ and SNQ (**7a**), AQ and FB (**7b**), and SNQ and FB (**7c**) as a function of Experiment, as indicated by circle (Experiment 1) and triangle (Experiment 2) data points. Pearson correlation coefficients are reported for each Experiment in the Results sections.

Figure 6 shows the overall interparticipant mean correct RTs for each trial type and as a function of gender.

#### Social gaze vs. Eye outline motion

A repeated measures ANOVA with cue validity (Social gaze vs. Eye outline motion) and cue-target interval (100, 375, 650, and 925 ms) revealed that participants were always faster to respond to targets that coincided with the social gaze cue relative to targets that coincided with the eye outline motion cue [cue validity; F(1,63) = 84.7, p < 0.0001]. A typical foreperiod effect emerged as well [cue-target interval; F(3,189) = 171.2, p < 0.0001]. A two-way interaction between cue validity and cue-target interval [F(3,189) = 5.1, p < 0.01] indicated that the difference between social and nonsocial cuing magnitudes increased as the cue-target time lengthened. When gender was included as a between-subjects variable, an interaction between cue validity and gender [F(1,62) = 4.1, p < 0.05] suggested that relative to females, males overall responded slower for targets coinciding with the eye outline motion, showing larger magnitudes of orienting (all other effects involving gender, Fs < 1). Follow up t-tests indicated that both genders overall responded faster for Social Gaze relative to Outline Motion trials (both ts > 5, ps < 0.001), and were overall equally fast to respond to both types of trials (both ts < 1, ps > 0.7).

#### Mouth motion

Aside from a reliable foreperiod [cue-target interval, F(3,189) = 164.6, p < 0.001], a repeated measures ANOVA with cue validity (Mouth motion vs. Invalid) and cue-target interval indicated no reliable effects or interactions (cue validity F < 1; cue validity x cue-target interval, F = 1). Analysis of gender suggested that male participants showed a trend towards responding faster for targets at invalid rather than valid target locations [i.e., those not coinciding with a motion cue; F(1,62) = 3.8, p = 0.055], which is reminiscent of an inhibition effect often observed during nonsocial orienting^23^,^79^, and dovetails with Bayliss *et al*.^42^ who also found no gender differences during nonsocial orienting. No other effects involving gender were reliable (all Fs < 1.1, ps > 0.3).

We also examined the data using an omnibus repeated measures ANOVA with Trial type (i.e., Social gaze, Eye outline motion, Mouth motion, Invalid) and Cue-target interval (100, 375, 650, and 925 ms) included as variables. The results were the same as those reported above, with targets coinciding with the Social gaze cue overall facilitated relative to all other trial types [F(3,189) = 27.2, p < 0.001; follow-up two-tailed paired t-tests all ts > −4.6, ps < 0.001].

Thus, surprisingly, our results revealed little overall influence of motion cues. When in competition with the social gaze cue, participants were always faster to detect the targets cued by gaze relative to targets cued by eye outline motion. When a motion cue was presented by itself, no reliable differences in responses emerged. Next, we examined if these effects varied as a function of average and individual social competence.

### Average Social Competence

Table 1 shows the distribution of AQ, SNQ, and FB scores as a function of quartile score ranks for Experiment 2. Overall average questionnaire scores did not differ between experiments (AQ, SNQ, FB, all ts < 1.5, ps > 0.1, two-tailed). The questionnaire data continued to relate predictably. AQ scores were negatively related to both SNQ and FB (r = −0.268, p < 0.05, and r = −0.248, p < 0.05, respectively) while SNQ and FB scores remained positively related (r = 0.456, p < 0.001). The composite social competence score was calculated using the same procedure as in Experiment 1. Participants with composite social competence scores falling between 3 and 7 were included in the low social competence group (N = 31; 15 males) while participants with composite social competence scores falling between 8 and 12 were included in the high social competence group (N = 33; 17 males).

Two mixed effects ANOVAs with social competence (low vs. high; between-subjects), cue validity, and cue-target interval (within-subjects) were run. The first was conducted for trials in which the social gaze cue occurred together with the nonsocial eye outline motion cue. The second ANOVA was conducted for trials in which the mouth motion occurred by itself. Unlike Experiment 1, no differences in either the social or motion cuing effect magnitudes emerged as a function of the participants’ average social competence. Neither ANOVA returned any reliable interactions involving cue validity and social competence (social competence x cue validity, both Fs < 1, ps > 0.4; social competence x cue validity x cue-target interval, both Fs < 1.4, ps > 0.25). Social gaze cues continued to produce reliable cuing effects overall [cue validity; F(1,62) = 83.4, p < 0.0001] which increased in magnitude with the lengthening of cue-target time [F(3, 186) = 5.0, p < 0.05]. No overall cuing effects of motion were reliable [cue validity, F < 1]. Thus, the magnitudes of social cuing did not vary with average social competence when social gaze cues were presented alongside motion cues.

### Individual Differences in Social Competence

Finally, we assessed whether any differences in social and nonsocial motion cuing related to individual differences in social competence. The data from three outliers were omitted from the correlation and regression analyses. In two separate multiple linear regressions, we entered participants’ gender, and their AQ, SNQ, and FB scores as predictors of the social cuing (average Eye outline motion RT – average Social gaze RT) and the isolated motion cuing magnitudes (average Invalid RT – average Mouth motion RT). The regression carried out on the social cuing magnitudes returned a reliable overall model with about 11% of the variance accounted for by the variables (R^2^ = 0.11, F(4, 56) = 2.8, p < 0.05). AQ emerged as a significant predictor (β = 0.344, t = 2.6, p < 0.05) with participants higher in AQ scores showing larger differences between targets coinciding with the social cue versus those coinciding with a motion cue. The regression analysis carried out on the isolated motion effect magnitudes was not reliable (R^2^ = 0.06, F(4, 56) = 1.9, p > 0.1).

**Figure 8:**
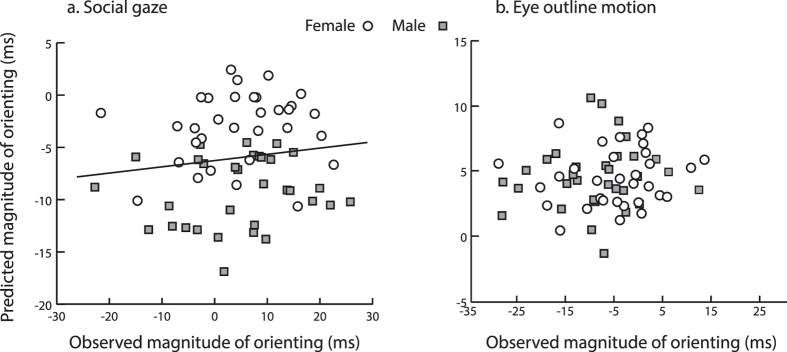
Experiment 2 Regression Results. Figure 8a shows the scatterplot along with the estimated linear fit displaying the relationship between individual participants’ observed (x-axis) and associated predicted (y-axis) magnitudes of social gaze advantage. Figure 8b shows the scatterplot displaying the relationship between individual participants’ observed (x-axis) and associated predicted (y-axis) magnitudes of eye outline motion advantage. Participants’ gender is marked by circle (Female) and square (Male) data points.

To understand whether the increased magnitude between social gaze and eye outline motion trials reflected speeding up of responses towards targets coinciding with the gaze cue or slowing down of responses for targets coinciding with the eye outline motion cue, we contrasted each Social gaze and Eye outline motion condition against the common Invalid condition. Two additional regressions were performed. The first examined variations in the magnitude of the social gaze cuing advantage (average Invalid RT – average Social gaze RT) and the second examined variations in magnitude of the eye outline motion cuing advantage (i.e., average Invalid RT – average Eye Outline motion RT). As before, gender and individual social competence scores were entered as predictors in each analysis. The regression carried out on the social gaze cuing advantage was once again reliable, with 11% of the variance accounted for by the variables (R^2^ = 0.115, F(4, 56) = 2.9, p < 0.05). AQ emerged as a reliable predictor (β = −0.333, t = −2.5, p < 0.05) with gender also closely approaching significance (β = −0.228, t = −1.9, p = 0.07). Individuals high in social competence showed increased social gaze cuing advantage relative to those with low social competence. The second model was not reliable (R^2^ < 0.1, F < 1, p = 0.6). Figure 8 shows the relationship between individual participants’ observed and predicted magnitudes of Social gaze cuing and Eye outline motion cuing advantage as a function of gender.

These final analyses tease apart whether the relationship between orienting magnitudes and social competence was driven by the social gaze cue advantage or the eye outline motion cue advantage. Individuals with higher social competence displayed larger response facilitation for targets coinciding with the social gaze cue relative to individuals with low social competence. And while a similar pattern of results emerged for males and females, overall female participants showed a marginally larger social cuing advantage.

## Discussion

These data once again suggest that individuals higher in social competence preferentially orient attention in response to social gaze over concomitant motion information. In Experiment 2 this finding emerged only when the magnitude of social cuing was examined as a function of individual differences in social competence, and not in group-based analyses, which showed overall facilitation for targets cued by social gaze. These data conceptually dovetail with those reported by Bayliss and colleagues^17^, who also demonstrated the superiority of social gaze over motion cues in typically developing adults. Extending those results, our data indicated that in addition to gross motion signals, local isolated mouth motion signals had little effect on attentional orienting both overall and as a function of average and/or individual social competence. Both of these results likely reflect a generally powerful overall attentional bias within the typical population for social relative to nonsocial information. That is, since our participants displayed normative social function, it is expected that any modulating effects of the nonsocial low-level cues on social gaze cuing would be more nuanced relative to what one would expect to observe in atypically functioning groups. In sum, the data from Experiment 2 indicated preferential attentional biasing to social gaze cues in individuals with high social competence.

Together, the results from our two experiments show that the presence of task-irrelevant luminance and motion features leads to a reduction in the magnitudes of gaze cuing in individuals with low social competence. While we interpret this result as indicating an attentional bias towards perceptual rather than social information in participants low in social competence, it has been suggested to us that alternatively these data may be accounted for by participants’ level of distractibility. There are two points worth considering here. First, among other symptoms, low social competence may include increased distractibility, as individuals with autism often display heightened attention to detail. Our results are not inconsistent with this notion, as higher total AQ score, denoting overall lower social competence was reliably related to an increase in the ‘attention to detail’ AQ subscale (Experiment 1; r = 0.515, p < 0.001; Experiment 2; r = 0.514, p < 0.001). However, critically, the larger construct of low social competence is also marked by a reduction in core autism-like symptoms, including poor social skills, behavioral issues, and communication dysfunctions. All of these AQ subcomponents also correlated highly with total AQ scores in both experiments (all rs > 0.54, ps < 0.001) highlighting the key contributing effects of core social deficits to the total AQ score beyond the ‘attention to detail’ component alone. Thus, while low social competence may be partially marked by increased attention to detail, this factor alone does not appear to be sufficient to fully account for the effects of the larger construct of lowered social function. Second, if the general distractibility explanation held, one would also expect the data to dissociate based on perceptual changes in the task rather than participants’ social competence. Our results did not support this prediction. Instead, they indicated that low social competence was reliably associated with magnitudes of gaze cuing regardless of whether there was a perceptual change or not in Experiment 1, and further, that low social competence was uniquely related to the magnitude of social but not motion cuing in Experiment 2. Therefore, while participants’ distractibility may be a feature of low social competence (see also recent discussions between commonalities and differences between ASD and ADD/ADHD^80^), prior data and our present results are not consistent with the hard alternative distractibility explanation.

### General Discussion

The main goal of this study was to investigate whether gaze cuing related to a broadly defined social competence construct within typically developing individuals. Based on past data collected with individuals with autism^45^,^46^, we hypothesized that typical persons low in social competence may perceive and attend to their visual environment differently than those high in social competence. Specifically, we reasoned that instead of accessing social information conveyed by social cues directly (i.e., the social reading hypothesis), individuals low in social competence, similarly to persons with autism, might orient their attention in the direction of social gaze because of the idiosyncratic low-level perceptual changes that often coincide with gaze shifts in everyday life (e.g., changes in the background, blinks, head and gaze actions; i.e., the feature correspondence hypothesis). If so, individuals low in social competence should be preferentially biased to attend to such low-level cues rather than the social information conveyed by gaze. Our data supported this hypothesis. In two Experiments, we examined the susceptibility of gaze cuing to feature-based perceptual changes (Experiment 1) and motion cues (Experiment 2), both of which were manipulated independently and orthogonally to social gaze. Across both studies we found that individuals low in social competence displayed reduced gaze cuing effects when the task contained irrelevant perceptual fluctuations. This stood in contrast to participants high in social competence whose gaze cuing effects remained preferentially biased towards social cues. Overall, the luminance-based changes in Experiment 1 appeared to exert stronger modulating effects relative to the motion cues in Experiment 2, while more stable social individual characteristics, specifically gender and number of autism-like traits (i.e., AQ score) emerged as stronger predictors of the social gaze cuing magnitudes in both experiments. It is important to note that these data do not merely reflect the gender composition of social competence groups. Specifically, we found that the average scores on each of the three questionnaires did not differ statistically across the two genders (AQ: t(126) = −1.9, p = 0.06; SNQ: t(126) = 1.1, p > 0.2; FB: t(126) < 1, p > 0.8; independent-samples, two tailed) while gender did not reliably interact with social competence and cue validity in either experiment (all interactions involving gender, social competence, and cue validity Experiment 1: all Fs < 1, ps > 0.3; Experiment 2: all Fs < 1, ps > 0.4).

Taken together, these data reveal strong links between perceptual processes, attentional biases, and a range of social functioning. Specifically, they suggest that individuals low in social competence appear to be preferentially biased to utilize mechanistic or feature-based access to social information. In contrast, those high in social competence appear to be strongly biased towards reading social information directly. As such, these results provide some of the first insights into the reasons for why gaze cuing magnitudes may fluctuate within a typical population, and carry three general implications.

The first concerns the links between social competence and basic cognitive operations. It is well accepted that inferring complex social and mental states requires basic perceptual and gaze orienting abilities^26^. Research suggests that humans utilize gaze to relay and understand a range of social signals including intentions, emotions, actions, and mental states of others^27^,^53^,^81^. The processes of accessing, reading, and attributing social information necessitate sophisticated underlying perceptual mechanisms that distinguish between social and nonsocial bits of information in the surrounding^82^. However, both types of information play a role in the development of a full range of social expertise. Gaze following in typical development initially depends on following low-level perceptual changes that coincide with social cues^67^,^69^,^83^. Within the first year, however, infants begin to access the social content of gaze directly^84^,^85^ and come to understand the psychological meaning of gaze only around 3 years of age^68^,^69^. Our data suggest that the performance of typically functioning adults with low social competence may resemble the performance of young infants who preferentially attend to nonsocial visual transients over social cues. It is possible that a prolonged reliance on the mechanistic access to social information may hinder socialization processes, contributing in turn to the development of reduced social competence. The opposite direction of influence is equally plausible, in that early instances of low social competence may facilitate prolonged patterns of preferential orienting towards low-level visual features rather than social information. Fundamentally however, the result of this intricate perceptual interplay appears to be consistent with the hypothesis of mechanistic access to social information in persons with reduced social competence (i.e., the feature correspondence hypothesis). That is, similarly to persons with autism, typically developing individuals with lower social competence appear to be biased towards attending to changes in environmental perceptual features relative to social information. This conclusion draws links between the variability in social function within typical and atypical populations and dovetails with recent studies indicating strong prospective links between the atypicalities in early perceptual development and severity of later-developed autism-spectrum phenotypes^64^. Future investigations aimed at understanding the relationship between perceptual styles, attentional preferences, attribution of social meaning to gaze, and developmental trajectories of social competence will be able to shed more light on the directionality of the relationship between perceptual processing, gaze cuing, and social competence.

The second implication of our results is that they provide a first comprehensive assessment between a broad construct of social competence and basic gaze cuing abilities. Our results revealed that the scores on the three different social function measures related predictably, and overall accounted for up to 30% of the variance in the magnitudes of the gaze cuing effects. This analysis both serves to establish a normative standard regarding the links between different facets of social competence, and to highlight our main finding that the experimental measure of gaze cuing relates meaningfully to the broad construct of social competence within the typical population. Our analyses also revealed that more stable characteristics like gender and AQ scores were stronger predictors of the magnitudes of gaze cuing relative to the more dynamic measures of actual and perceived social network size. There are at least two possibilities for why this may be the case. One reason could be the nature of the cuing task, which is purported to experimentally index stable and situationally invariant attentional processes. This suggests that measures of gaze following obtained using more contextually-situated and ecologically valid procedures (e.g. refs 65 and 86) should relate to dynamic scores of social functioning more strongly. Another potential reason for why stable traits were more strongly related to gaze cuing could lie in the efficacy of the social network size measures in accurately reflecting the extent of an individual’s social network. It is possible that in addition to one’s core social network, SNQ and FB questionnaires also reflect extended and contextually mediated social circles. Specifically, the SNQ yields the number of individuals a participant reports as having had social interactions with during the last month. As such, this score might include situationally facilitated interactions that depend on an individual’s current environment or particular time of year (e.g., holidays). Consequently, depending on the time of assessment, these questionnaires might return variable estimates of the core social network size. A similar argument may be made for the number of Facebook friends, whereby online social networks may also contribute to a reduction in social function through, for example, an increase in depressive symptoms^87^. Future investigations could examine factors that influence fluctuations in the estimates of social network size over the course of a longer period of time to better understand if and how one’s core social network size may relate to basic social cognitive functions.

Finally, this work provides important methodological bridges between traditional cognitive approaches, which seek to reveal commonalities in cognitive functions across individuals^23^ and a burgeoning literature on social attention in real world interactions, which seeks to reveal factors that may modulate these basic operations within individuals and across situations^88^,^89^. Our work suggests that gaze cuing behavior operates across both basic and more complex social situations when an individual’s level of social competence along with their gender are taken into account. In doing so, our data provide some initial boundary conditions for future multi-level investigations of socio-cognitive function across simple experimental and complex real-world contexts.

In sum, here we traced what is purported to be a fundamental socio-cognitive mechanism, namely gaze following, through the levels of perceptual, cognitive, and social analyses. In two experiments we related participants’ variability in the magnitude of gaze cuing with individual characteristics (i.e., gender), social traits (i.e., number of autistic-like traits), and dynamic social behaviors (SNQ, FB). Our data indicate that the level of social competence within typically developing individuals is related to how they perceive and access social information in the environment.

## Additional Information

**How to cite this article:** Hayward, D. A. and Ristic, J. Feature and motion-based gaze cuing is linked with reduced social competence. *Sci. Rep.* **7**, 44221; doi: 10.1038/srep44221 (2017).

**Publisher's note:** Springer Nature remains neutral with regard to jurisdictional claims in published maps and institutional affiliations.

## Figures and Tables

**Table 1:** Social competence measures summaries for Experiments 1 and 2.

	*Autism Spectrum Quotient (AQ*)			*Social Network Questionnaire (SNQ*)			*Facebook (FB*)		
	Range	M	SD	Range	M	SD	Range	M	SD
**Experiment 1**									
Quartiles 1 & 2	17–31	21.2	3.7	9–34	23	6.6	0–450	266	132
Quartiles 3 & 4	5–17	12.4	3.6	34–73	49	12.2	450–1500	701	248
**Experiment 2**									
Quartiles 1 & 2	18–35	23	4.3	5–29	20	6.4	0–360	176	114
Quartiles 3 & 4	6–17	12.5	2.6	29–140	48	20.2	400–1800	838	366
